# Epigenetic reprogramming underlies efficacy of DNA demethylation therapy in osteosarcomas

**DOI:** 10.1038/s41598-019-56883-0

**Published:** 2019-12-30

**Authors:** Naofumi Asano, Hideyuki Takeshima, Satoshi Yamashita, Hironori Takamatsu, Naoko Hattori, Takashi Kubo, Akihiko Yoshida, Eisuke Kobayashi, Robert Nakayama, Morio Matsumoto, Masaya Nakamura, Hitoshi Ichikawa, Akira Kawai, Tadashi Kondo, Toshikazu Ushijima

**Affiliations:** 1 Division of Rare Cancer Research, National Cancer Center Research Institute, 5-1-1 Tsukiji, Chuo-ku, Tokyo, 104-0045 Japan; 2 Department of Orthopaedic Surgery, Keio University School of Medicine, 35 Shinanomachi, Shinjuku-ku, Tokyo, 160-8582 Japan; 3 Division of Epigenomics, National Cancer Center Research Institute, 5-1-1 Tsukiji, Chuo-ku, Tokyo, 104-0045 Japan; 4 Department of Clinical Genomics, National Cancer Center Research Institute, 5-1-1 Tsukiji, Chuo-ku, Tokyo, 104-0045 Japan; 5 Department of Pathology and Clinical Laboratory, National Cancer Center Hospital, 5-1-1 Tsukiji, Chuo-ku, Tokyo, 104-0045 Japan; 6 Department of Musculoskeletal Oncology, National Cancer Center Hospital, 5-1-1 Tsukiji, Chuo-ku, Tokyo, 104-0045 Japan

**Keywords:** Chemotherapy, Bone cancer, Paediatric cancer, Sarcoma

## Abstract

Osteosarcoma (OS) patients with metastasis or recurrent tumors still suffer from poor prognosis. Studies have indicated the efficacy of DNA demethylation therapy for OS, but the underlying mechanism is still unclear. Here, we aimed to clarify the mechanism of how epigenetic therapy has therapeutic efficacy in OS. Treatment of four OS cell lines with a DNA demethylating agent, 5-aza-2′-deoxycytidine (5-aza-dC) treatment, markedly suppressed their growth, and *in vivo* efficacy was further confirmed using two OS xenografts. Genome-wide DNA methylation analysis showed that 10 of 28 primary OS had large numbers of methylated CpG islands while the remaining 18 OS did not, clustering together with normal tissue samples and Ewing sarcoma samples. Among the genes aberrantly methylated in primary OS, genes involved in skeletal system morphogenesis were present. Searching for methylation-silenced genes by expression microarray screening of two OS cell lines after 5-aza-dC treatment revealed that multiple tumor-suppressor and osteo/chondrogenesis-related genes were re-activated by 5-aza-dC treatment of OS cells. Simultaneous activation of multiple genes related to osteogenesis and cell proliferation, namely epigenetic reprogramming, was considered to underlie the efficacy of DNA demethylation therapy in OS.

## Introduction

Osteosarcoma (OS) is the most common malignant tumor of the bone in children and adolescents^1^. Primary OS frequently shows severe aneuploidy and the highest mutation frequency among all pediatric cancers, including Ewing sarcoma (EWS). The most commonly mutated genes in OS are tumor-suppressor genes *TP53* (47–90%) and *RB1* (29–61%)^2–4^. In spite of the paucity of mutated genes, the landscape of epigenetic alterations, namely aberrant DNA methylation, in primary OS is poorly understood^5^. Most previous studies on DNA methylation in OS focused on genes involved in cancer-related pathways, such as cell cycle regulation, apoptosis, cell proliferation, and differentiation^6–12^. One genome-wide study analyzed cell lines^13^, and another recent one analyzed a limited number of primary OS samples^5^.

While treatment of OS without metastases was advanced by introduction of multimodal therapies, including surgery combined with chemotherapy^1,14,15^, treatment of OS with metastasis or recurrent tumors has not changed over the past 30 years, with a poor survival rate of less than 30%^1,14,16–18^. As a potential clue to novel therapeutic strategies, treatment of OS xenografts with DNA demethylating drugs, including 5-aza-2′-deoxycytidine (5-aza-dC) and 5-azacytidine (5-aza-CR), showed promising results^6,10,19^. However, molecular mechanisms underlying the therapeutic efficacy of DNA demethylating drugs have not been fully elucidated. As a mechanism of action of DNA demethylating drugs, reactivation of specific tumor-suppressor genes used to be hypothesized, and this was also proposed for OS^5,6,11,12,19–21^. However, recent studies in other types of cancers highlighted the importance of simultaneous re-activation of multiple tumor-suppressor genes, namely epigenetic reprogramming^22–24^, and emphasized the importance of low dose and prolonged exposure^25–27^. In addition, sensitization of cancer cells to immunotherapy and chemotherapy is proposed as an important mechanism of action of DNA demethylating drugs^28–31^.

In this study, we aimed i) to confirm the efficacy of DNA demethylation therapy in OS using a low dose and prolonged exposure, and ii) to reveal the mechanism of how DNA demethylation therapy exerts its efficacy in OS.

## Results

### Therapeutic efficacy of DNA demethylation therapy

**Figure 1:**
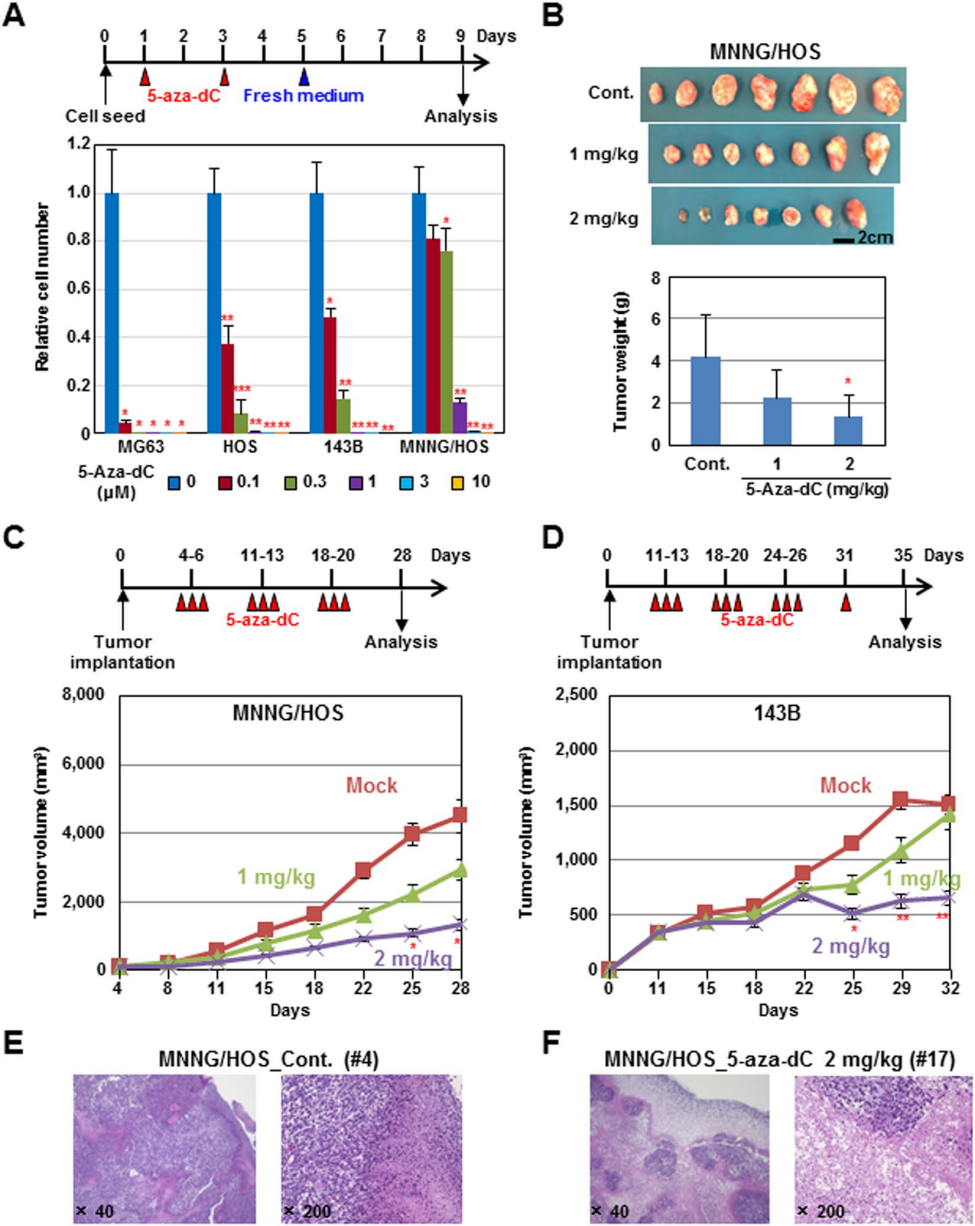
Effect of DNA demethylation therapy against OS cell lines. (**A**) Experimental protocol of drug treatment *in vitro*. Four OS cell lines (MG63, HOS, 143B, MNNG/HOS) were treated with 5-aza-dC (0, 0.1, 0.3, 1, 3, and 10 μM) for 4 days, and the cell number was counted on day 9. Cell growth was significantly suppressed by treatment with low doses of 5-aza-dC (0.1–0.3 μM). (**B–D**) The *in vivo* effect of the DNA methylation therapy. 5-Aza-dC was intraperitoneally administered for 3 days followed by drug holidays of 4 days, and the treatment was repeated for three or four cycles (top of C, and D). The treatment with 5-aza-dC reduced tumor volume (**B–D**) and weight (**B**) of both MNNG/HOS and 143B xenograft tumors. (**E,F**) Pathological analysis of the xenografts. In the 5-aza-dC-treated tumors, marked tumor cell necrosis was observed (**E,F**). *P < 0.05, **P < 0.01, ***P < 0.001.

The growth inhibitory effect of 5-aza-dC on OS cells was first analyzed using a protocol that exposed cells to 5-aza-dC for four days and implemented 4-day culture under fresh medium. This “long-term” protocol is known to exert maximum DNA demethylating effects^25–27^. *In vitro* growth of three of four OS cell lines, MG63, HOS, and 143B, was strongly inhibited by the 5-aza-dC treatment in a dose-dependent manner (Fig. 1A).

*In vivo* therapeutic effect was further analyzed using MNNG/HOS and 143B xenograft tumors, which are frequently used in animal models. MNNG/HOS tumor volume was suppressed to 65.0% and 29.3% of mock-treated tumors by administration of 1 and 2 mg/kg, respectively, of 5-aza-dC for three weeks (Fig. 1B,C). 143B tumor volume was also suppressed to 94.0 and 43.1% by administration of 1 and 2 mg/kg, respectively, of 5-aza-dC for four weeks (Fig. 1D, and Supplementary Fig. 1A). Necrosis in tumor tissues was increased by the 5-aza-dC treatment (Fig. 1E,F, Supplementary Fig. 1B,C), and the numbers of lung metastases tended to decrease to 97.4 and 57.1% of mock-treated mice by administration of 1 and 2 mg/kg, respectively, of 5-aza-dC, but without statistical significance (Supplementary Fig. 1D–F). These results showed that DNA demethylation therapy is effective for treatment of OS, in line with previous reports^7,9,10,32^.

### Genome-wide DNA methylation profiles in osteosarcomas

**Figure 2:**
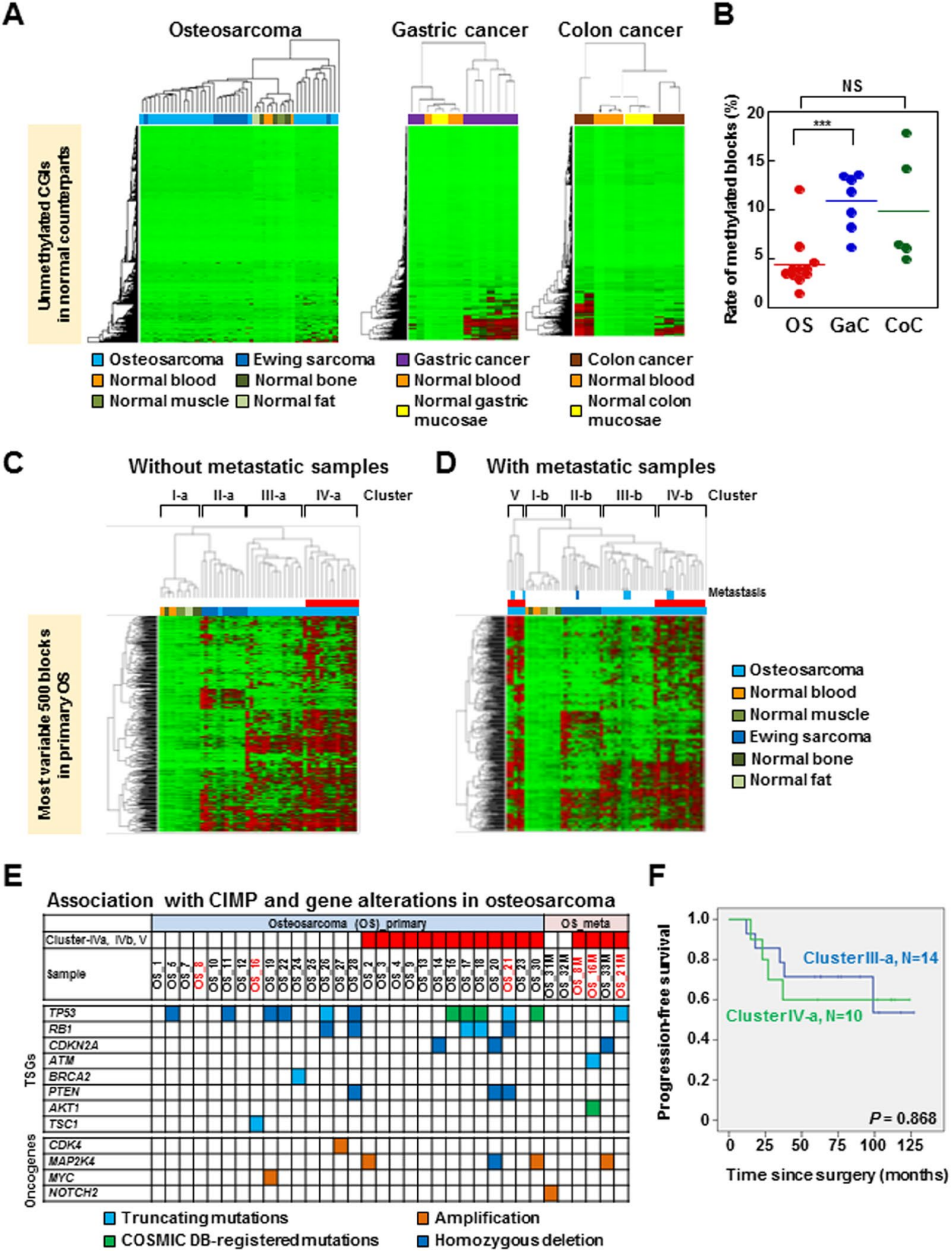
The Genome-wide DNA methylation profiles of primary OS. (**A**) Unsupervised hierarchical clustering analysis using normally unmethylated CGIs (47,759 genomic blocks) of primary OS (n = 28), Ewing sarcoma (EWS) (n = 11), normal whole blood (n = 3), muscle (n = 3), fat (n = 2), and bone (n = 3) samples. Ten OS and one ES were classified into a cluster with larger numbers of methylated CGIs. The remaining 18 OS and 10 EWS clustered together with normal samples with limited numbers of methylated CGIs. Gastric cancer (n = 8) and colorectal cancer (n = 5) (41,452 genomic blocks) were similarly clustered for comparison. (**B**) Fraction of methylated CGIs in OS with a relatively large number of methylated CGIs, CIMP(+) gastric cancer, and colon cancer. The fraction was much smaller in the OS than in the CIMP(+) gastric cancer. (**C**) Unsupervised hierarchical clustering analysis of primary OS samples using the 500 most variable genomic blocks. Primary OS samples were classified mainly into two clusters, and one cluster (cluster IV-a) had a larger number of methylated genomic blocks. (**D**) Unsupervised hierarchical clustering analysis of primary and metastatic OS samples. The 500 most variable genomic blocks used in (**C**) were utilized, and four of six metastatic OS samples were clustered with primary OS samples in cluster IV-a (clusters IV-b and V). (**E**) Distribution of somatic mutations in the OS samples in clusters IV-a and cluster III-a. There was no association between the methylation clusters and mutation frequency of a specific gene. Tumors in red are the pairs of primary and metastatic tumors from same patients (the pairs are OS_8 and OS_8M, OS_16 and OS_16M, OS_21 and OS_21M). (F) Kaplan-Meier curves of progression-free survival (PFS) for 28 primary (M0) OS patients according to their methylation clusters. There was no significant difference (*P* = 0.868).

Genome-wide DNA methylation profiles were then analyzed by a methylation beadarray of i) 34 OS surgical specimens (28 primary and six metastatic) from 31 patients, ii) 11 EWS surgical specimens (ten primary and one metastatic) from 11 patients, and iii) 11 normal tissue samples (three blood, three muscle, three bone, and two fat samples). To focus on DNA methylation of genomic regions stably maintained, we focused on CpG islands (CGIs)^33^, and multiple CpG probes in a region were assembled into a genomic block. In order to compare the CGI methylation profile of OS with that in other cancers, we first analyzed genomic blocks randomly selected from those in CGIs. Unsupervised hierarchical clustering analysis showed that primary OS can be classified into two clusters. Ten OS samples with larger numbers of aberrantly methylated genes formed one cluster with one EWS sample, and 18 OS samples with smaller numbers formed another cluster with the other 10 EWS samples and normal control samples (Fig. 2A). However, the number of aberrantly methylated genes in OS samples in the former cluster was much smaller than that in the CGI methylator phenotype (CIMP) (+) gastric and colon cancers, in which the presence of CIMP is well established^34,35^ (Fig. 2B).

Then, using the 500 most variable genomic blocks, unsupervised hierarchical clustering analysis was conducted. Primary OS samples were classified mainly into two clusters (clusters III-a and IV-a; Fig. 2C); one having a larger number of methylated genomic blocks and the other not. When six OS and one Ewing metastatic samples were added to the cluster analysis using the 500 most variable genomic blocks, four of the six metastatic OS samples were clustered with primary OS samples in clusters IV-b and V, derived from cluster IV-a (Fig. 2D). However, between cluster III-a and cluster IV-a, there were no differences in somatic mutations of tumor-suppressor genes oncogenes (Fig. 2E) or in disease-free survival (*P* = 0.868) (Fig. 2F, and Supplementary Table 3). These results showed that the biological significance of the methylation clusters in the OS samples was unclear.

### Aberrant DNA methylation of osteogenesis-related genes in OS

**Figure 3:**
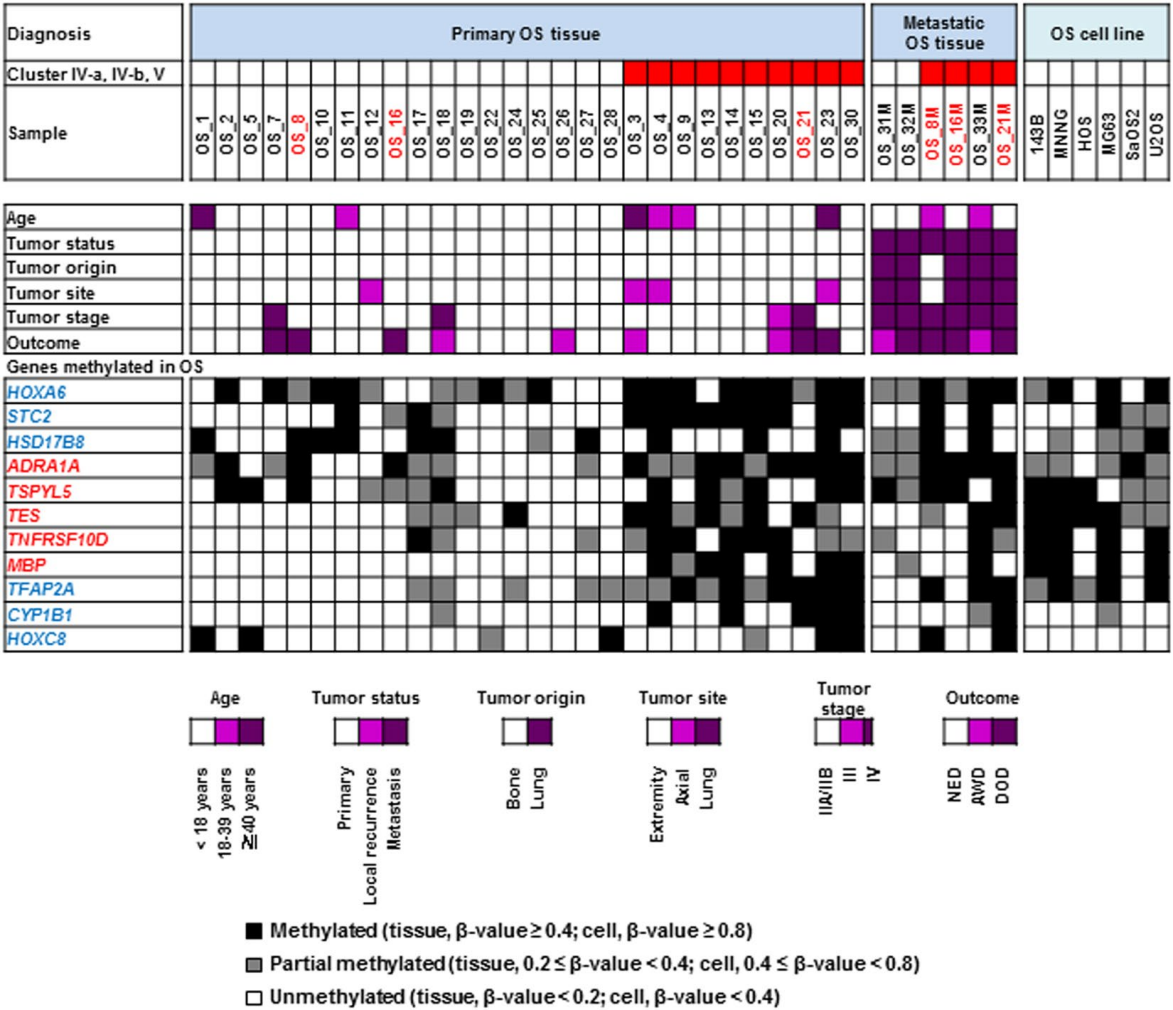
Methylation statuses of the potentially methylation-silenced osteogenesis- / chondrogenesis-related genes and tumor-suppressor genes among the 28 primary OS tissues, six metastatic OS tissues, and six OS cell lines. Genes were selected based on the result of gene ontology analysis. Genes related to osteogenesis were frequently methylated in OS regardless of their methylation clusters. Tumors in red are the pairs of primary and metastatic tumors from same patients (the pairs are OS_8 and OS_8M, OS_16 and OS_16M, OS_21 and OS_21M). Genes in blue are osteogenesis- or chondrogenesis-related genes, and genes in red are tumor-suppressor genes. OS, osteosarcoma; NED, no evidence of disease; AWD, alive with disease; DOD, dead of disease.

To explore what genes were inactivated by aberrant DNA methylation, we focused on methylation of CpG sites within 200 bp from a transcription start site (TSS200) in a CGI. We searched for genes i) whose TSS200 CGIs were unmethylated in the bone and methylated in OS and ii) which were expressed in mesenchymal stem cells and osteoblasts. Gene ontology analysis revealed that genes involved in hormone metabolism (*CYP1B1*, *STC2*, *HSD17B8*), neurological function (*CYP1B1*, *TFAP2A*, *ADRA1A*, *MBP*), and skeletal system morphogenesis (*HOXC8*, *HOXA6*, *TFAP2A*) were enriched among the potentially methylation-silenced genes in OS (Table 1). Among these genes, six (*HOXA6*, *STC2*, *HSD17B8*, *TFAP2A, CYP1B1*, and *HOXC8*) were reported to be osteogenesis-/chondrogenesis-related genes, and five (*ADRA1A*, *TSPYL5*, *TES*, *TNFRSF10D*, and *MBP*) were reported to be tumor-suppressor genes (Fig. 3). These results showed that genes related to osteogenesis are frequently methylation-silenced in OS.

**Table 1:** Functional annotation of genes methylation-silenced in primary OS.

Subtypes	Term	*P*-value	Genes
**Osteosarcoma**	Hormone metabolic process	**0.018**	*CYP1B1, STC2, HSD17B8*
	Estrogen metabolic process	**0.032**	*CYP1B1, HSD17B8*
	Neurological system process	**0.049**	*CYP1B1, TFAP2A, ADRA1A, MBP*
	Skeletal system morphogenesis	0.051	*HOXC8, HOXA6, TFAP2A*
	Sensory perception	0.081	*CYP1B1, TFAP2A, MBP*
	Regulation of cell proliferation	0.085	*CYP1B1, TNFRSF10D, TFAP2A, ADRA1A, TSPYL5, TES*
	System process	0.089	*DYSF, CYP1B1, TFAP2A, ADRA1A, MBP*
	Negative regulation of cell proliferation	0.093	*CYP1B1, TFAP2A, ADRA1A, TES*

DAVID functional annotation analysis was conducted using the genes with TSS200 CGI expressed in mesenchymal stem cell (MSC)/osteoblast (OB) as a background (n = 4,338). Genes methylated (β-value ≥ 0.4) in more than 5 of 28 primary OS tissues (M0) are listed.

### Reprogramming of methylation-silenced genes by DNA demethylation therapy

**Figure 4:**
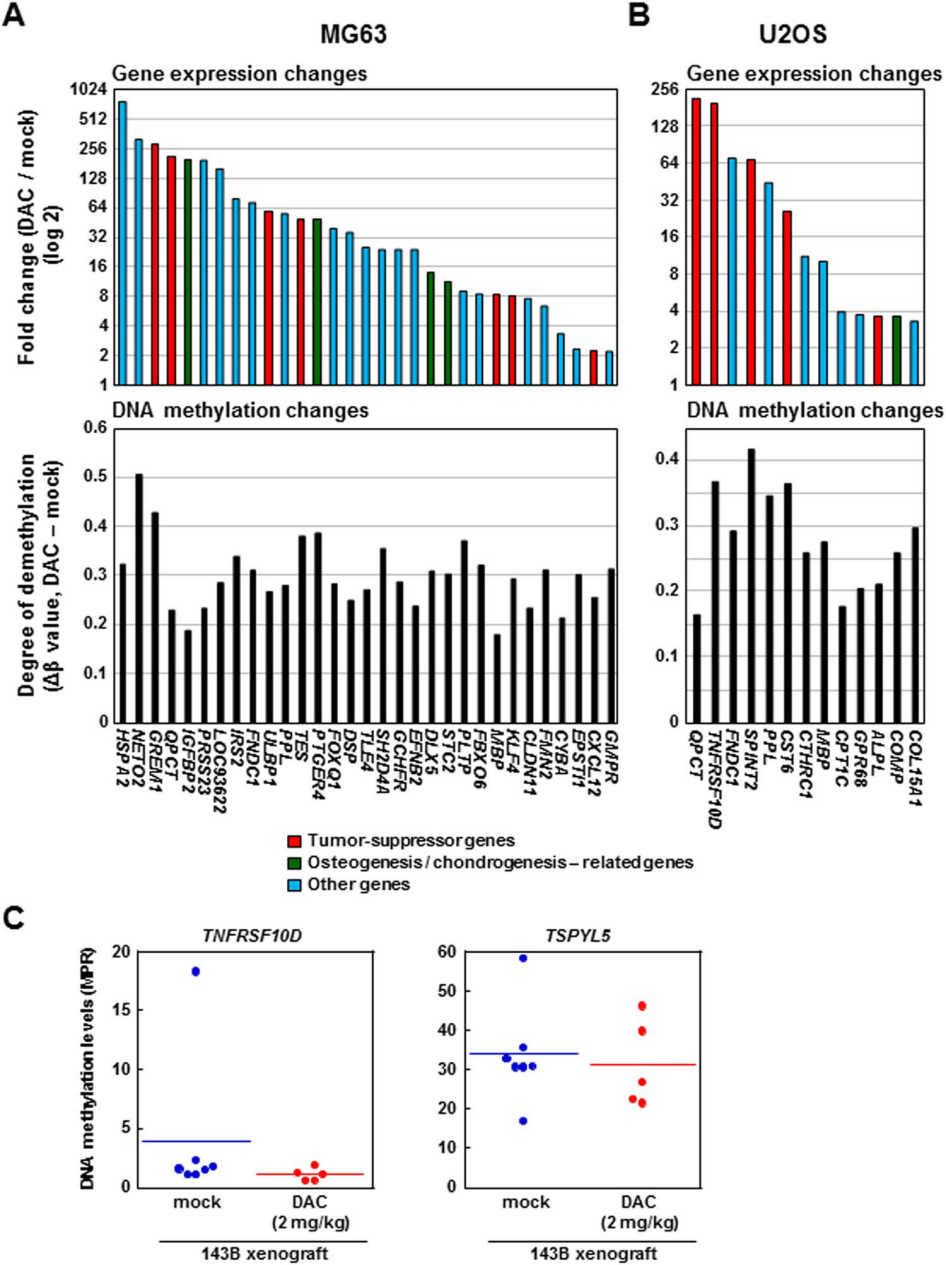
Identification of methylation-silenced genes by chemical genomic screening. Two OS cells (MG63, U2OS2) were treated with 1 µM of 5-aza-dC, and genes up-regulated compared with mock-treated cells were searched for using a microarray. We focused on 2,226 genes, (i) whose TSS200 CGIs were unmethylated in the normal tissue and methylated in OS, (ii) which were expressed in mesenchymal stem cells and osteoblasts, and (iii) whose genes expression changes could be evaluated by microarray analysis. Among the 2,226 genes, 31 and 13 genes were upregulated two-fold or more (signal log ratio > 2) in (**A**) MG63 and (**B**) U2OS, respectively. These genes contained tumor-suppressor (in red letters) and bone or cartilage formation-related genes (in green letters), whose function has been already reported. (**C**) DNA methylation changes by 5-aza-dC treatment in 143B xenograft tumors. DNA methylation levels of *TNFRSF10D* and *TSPYL5* were reduced by the treatment.

To identify methylation-silenced genes in MG63 and U2OS cells, the cells were treated with 5-aza-dC, and genes whose expression was induced were searched for by microarray analysis. Thirty-one genes in MG63 and 13 in U2OS were up-regulated at two-fold or more by 5-aza-dC treatment (Fig. 4A,B). DNA methylation levels of promoter CpG islands of these genes were reduced, ranging from 18 to 51% in MG63 and 17 to 42% in U2OS (Fig. 4A,B). Among the 31 genes in MG63, six and four genes were reported to be tumor-suppressors and be related to bone or cartilage formation, respectively (Supplementary Tables 4 and 5). Three of the 31 genes, *TES*, *STC2*, and *MBP*, were methylated in 6–11 of the 28 primary OS samples. Among the 13 genes in U2OS, five and one genes were reported similarly. Two of the 13 genes, *TNFRSF10D* and *MBP*, were methylated in 6 of the 28 primary OS samples. In the xenograft tumors, DNA methylation levels of *TNFRSF10D* and *TSPYL5* were decreased by the 5-aza-dC treatment (Fig. 4C). These results suggested that the demethylation of methylation-silenced tumor-suppressor and osteo/chondrogenesis-related genes underlies the therapeutic effect of DNA demethylation therapy.

### Epigenetic analysis of clonal evolution patterns of OS

**Figure 5:**
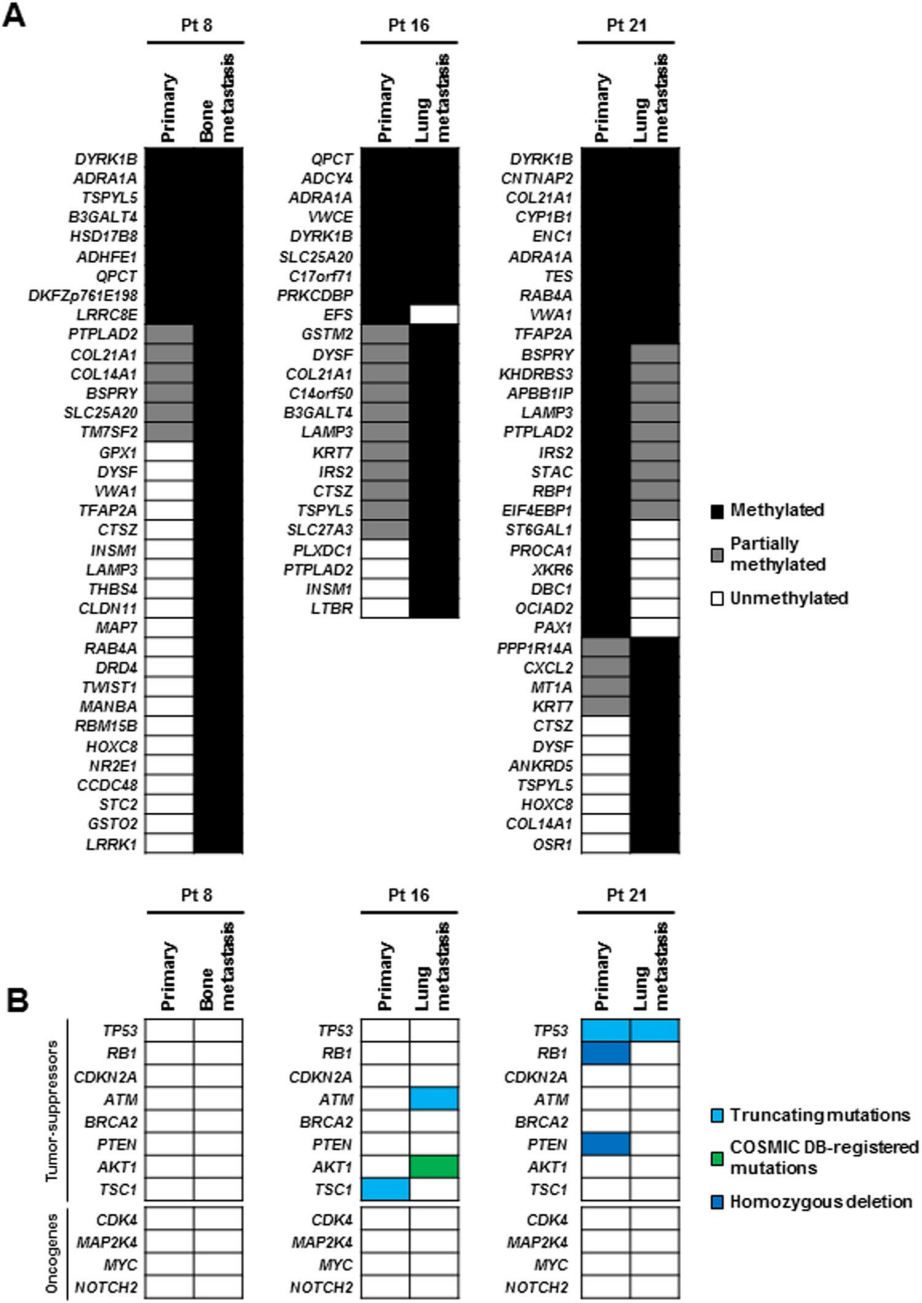
Molecular evolution of OS based upon DNA methylation (**A**) and gene mutations (**B**). (**A**) Shared and unique DNA methylation between three pairs of primary and metastatic tumors was analyzed. We analyzed 4,327 genes (i) whose TSS200 CGIs were unmethylated in the normal tissue and methylated in OS, and (ii) which were expressed in mesenchymal stem cells and osteoblasts. Two metastatic tumors (Patients #8 and #16) showed a pattern of clonal evolution, one tumor from Patient #21 showed a pattern of parallel evolution. (**B**) Genomic alterations using NCC Oncopanel v4 between three pairs of primary and metastatic tumors were analyzed. This genetic analysis did not reveal the evolutionary process of distant metastasis.

Having the DNA methylation profiles of primary and metastatic OS, clonal evolution of OS was analyzed as in previous reports^36,37^. Metastatic tumors of patient 8 (OS_8M) and patient 16 (OS_16M) had aberrantly methylated genes (β ≥ 0.40) in addition to those detected in their corresponding primary tumors (Fig. 5A). On the other hand, a metastatic tumor of patient 21 (OS_21M) had only a fraction of genes aberrantly methylated in its primary tumor, and had genes uniquely methylated (private methylation) in the metastatic tumor. These results indicated that the metastatic tumors of patients 8 and 16 have evolved clonally while that of patient 21 had a common ancestor lesion with the primary tumor. Due to the paucity of somatic mutations, speculation on molecular evolution was not possible based upon somatic mutation profiles (Fig. 5B).

## Discussion

Simultaneous activation of multiple tumor-suppressor and osteo/chondrogenesis-related genes, namely epigenetic reprogramming, was indicated to underlie the efficacy of DNA demethylation therapy in OS. We were able to confirm that several methylated genes, such as *STC2* (known as an osteogenesis related gene)^38^, *TES*, and *TNFRSF10D* (known as a tumor-suppressor gene)^39,40^, were demethylated and reactivated with demethylation therapy. Therefore, epigenetic reprogramming of tumor-suppressor and osteo/chondrogenesis-related genes was considered to be the major mechanism underlying the therapeutic efficacy against OS. Since two DNA demethylating drugs, decitabine (5-aza-dC) and azacitidine, are already approved by the Food and Drug Administration (FDA) for the treatment of hematological malignancies^25^, the mechanism of action shown here provides a rational basis for DNA demethylation therapy in OS.

The DNA methylation profiles in OS were different from those in gastric and colorectal cancers because OS had much fewer methylated genes (Fig. 2A), and the number of methylated loci did not appear to have a prognostic impact or a different mutation profile (Fig. 2F). Among the six metastatic OS samples, four were clustered with primary samples in cluster IV-a, which had a relatively larger number of methylated genomic blocks (clusters IV-b and V). The four metastatic samples clustered with primary samples contained 44, 62, 72, and 88% of cancer cell fractions while the remaining two metastatic samples had relatively low cancer cell fractions (32 and 36%). Accordingly, these two metastatic OS samples were not clustered with primary samples with cluster IV-a.

Recently, phylogenetic reconstruction of intratumor genomic heterogeneity of cancer samples was used to reveal branched evolutionary tumor growth^36,37^. In addition to mutation patterns, the DNA methylation patterns were also used to capture such evolutionary processes of cancer cells already in colorectal cancers and prostate cancers^41,42^. We adopted DNA methylation patterns of CpG islands in promoter regions, which are known to have stable patterns^43^, to capture clonal evolution in three OS patients. Two OS patients who developed bone and lung metastasis 38 and 12 months after primary tumor resection, respectively, showed clonal evolution of the metastasis from the corresponding primary tumors. In contrast, one OS patient who developed lung metastasis at the time of initial diagnosis before treatment of the primary tumor had private methylation, and was considered to have a common ancestor lesion, showing a parallel evolutionary pattern.

## Conclusions

DNA demethylation therapy was effective against OS. Reactivation of tumor-suppressor genes and osteo/chondrogenesis-related genes was suggested to be the mechanism underlying the therapeutic efficacy of DNA demethylation therapy in OS.

## Methods

### Surgical specimens

The study was approved by the institutional review board at the National Cancer Center (2004–050). All experimental methods were carried out in accordance with relevant guidelines and regulations. Written informed consent was obtained from all patients. Fresh frozen surgical specimens of OS (34 tumors from 31 patients) and EWS (11 tumors from 11 patients) were obtained from patients who underwent biopsy or surgery before chemotherapy or radiotherapy at the National Cancer Center Hospital (Tokyo, Japan) between 1998 and 2013. These specimens were provided by the National Cancer Center Biobank (Tokyo, Japan), and clinical characteristics of all the patients are shown in Supplementary Table 1. Cancer cell fractions of OS samples were estimated by the profile of cancer-specific DNA methylation^44^, and ranged from 46 to 72% (58.0 ± 8.6%) in the primary OS samples in cluster IV-a, from 34 to 80% (53.5 ± 14.3%) in the primary OS samples in cluster III-a, and from 32 to 88% (56 ± 22.4%) in the metastatic OS samples. As normal counterparts, bone (n = 2), fat tissue (n = 2), muscle (n = 2), and lung tissue (n = 1) were obtained from patients who underwent surgery at the National Cancer Center Hospital in 2016.

### Cell lines and cell culture

Six human OS cell lines (HOS, MNNG/HOS, 143B, MG63, SaOS2, and U2OS) and four EWS cell lines (RD-ES, W-ES, A673, and SK-ES-1) were used in this study. MNNG/HOS, U2OS, RD-ES, A673, and SK-ES-1 were purchased from the American Tissue Type Culture Collection (Rockville, MD, USA). HOS, 143B, MG63, and SaOS2 were purchased from the RIKEN BioResource Center (Tsukuba, Japan). W-ES was kindly provided by Dr. Maeda M^45^. HOS, MNNG/HOS, 143B, MG63, U2OS, and A673 were maintained in DMEM, RE-ES and W-ES were in RPMI1640, SaOS2 and SK-ES-1 were in McCoy’s 5 A medium. Cells were tested for mycoplasma infection with the MycoAlert Mycoplasma Detection Kit (Lonza, Basel, Switzerland).

### Extraction of genomic DNA, RNA, and protein

Frozen tissues were crushed using a Multi-beads Shocker (Yasui Kikai, Osaka, Japan) with cooling by liquid nitrogen. Genomic DNA was extracted from crushed frozen tissues and cell lines using the standard phenol-chloroform extraction method. Total RNA was extracted from crushed frozen tissues using a miRNeasy Mini Kit (Qiagen, Hilden, Germany).

### *In vitro* 5-aza-2′-deoxycytidine treatment

OS cell lines were seeded (MG63, 1 × 10^4^; HOS, 2 × 10^3^; 143B, 2 × 10^3^; and MNNG/HOS, 1 × 10^3^ cells) in a 6-well plate on day 0, and treated with various concentrations (0.1, 0.3, 1, 3, 10 μM) of 5-aza-dC on days 1 and 3. To facilitate cell proliferation, which can result in efficient DNA demethylation^26^, the medium was replaced by a fresh one without 5-aza-dC on day 5, and cells were harvested on day 9. Cell numbers were counted using an automated cell counter (TC20TM, Bio-Rad Laboratories, Inc., CA, USA).

### DNA methylation beadarray analysis

Genome-wide DNA methylation profile was analyzed using an Infinium HumanMethylation450 BeadChip array (Illumina, San Diego, CA, USA), as described previously^35^. Among the 485,512 CpG sites, 473,961 CpG sites located on autosomes were used for the analysis, and these CpG sites were assembled into 292,361 genomic blocks according to their locations against TSSs and CGIs^34,46^ (probe annotation of Illumina Infinium HumanMethylation450 on human reference genome, hg19). The DNA methylation level of a CpG site was assessed by the β value from 0 (unmethylated) to 1 (fully methylated). That of a genomic block was evaluated using an average of the β values of the CpG sites within the block, since the use of an average β value can efficiently isolate densely methylated CpG islands, which are important for methylation-silencing^46^. A genomic block was defined as unmethylated (0–0.2), partially methylated (0.2–0.4 for tissues; and 0.2–0.8 for cancer cell lines), and methylated (0.4–1.0 for tissues; and 0.8–1.0 for cancer cell lines). DNA methylation data of esophageal cancers (n = 12), gastric cancers (n = 8), colon cancers (n = 5), their corresponding normal tissues [esophageal mucosa (n = 1, GSE77991)^47^; gastric mucosa (n = 3)^34^; colon mucosa (n = 3, GSE42752); and blood samples (n = 3)], normal bone (n = 1, GSE50192), normal lung (n = 10, GSE52401) were obtained from Gene Expression Omnibus (GEO).

### Cluster analysis

Unsupervised hierarchical clustering analysis was performed using R 2.15 [R Core Team (2012) R: A language and environment for statistical computing. R Foundation for Statistical Computing, Vienna, Austria. ISBN 3-900051-07-0, http://www.R-project.org/] with the Heatplus package [Alexander Ploner (2011) Heatplus: Heatmaps with row and/or column covariates and colored clusters, R package version 2.2.0] from Bioconductor^48^. The Euclidean distance was used as the distance function both for samples and genes. Due to the limitation in the calculation algorithm for the hierarchical clustering, 20,000 elements or less were used for the analysis.

### Analysis of genetic alterations

Somatic mutations and copy number alterations of 114 genes and fusions of 12 genes were analyzed by target sequencing using a customized panel, NCC Oncopanel v4 (Supplementary Table 2), as described previously^49^. Briefly, sequencing libraries were prepared using SureSelect XT reagent (Agilent Technologies), and paired-end sequencing (2 × 150 bp) was performed using a NextSeq sequencer (Illumina, San Diego, CA, USA). To detect mutations, copy number alterations, and gene fusions from the sequencing read data, we used an in-house program cisCall^50^. For SNP elimination, we used 1000 Genomes (http://www.1000genomes.org), ESP6500 (http://evs.gs.washington.edu/EVS/), Human Genetic Variation Database (http://www.genome.med.kyoto-u.ac.jp/SnpDB), Tohoku Medical Megabank Organization data (https://ijgvd.megabank.tohoku.ac.jp/), and in-house Japanese germline SNP data. For annotation of identified mutations, we used the ANNOVAR^51^ and COSMIC^52^ databases. Increases of ≥4-fold and decreases of <2-fold in read depth were considered as candidates for gene amplification and homozygous deletion, respectively.

### Analysis of gene expression

Genome-wide gene expression was analyzed by a SurePrint G3 Human Gene Expression 8 × 60 K v2 Microarray (Agilent Technologies, Santa Clara, CA). cRNA labeled with Cy3 was synthesized from 200 ng of total RNA using a Low Input Quick Amp Labeling Kit (Agilent Technologies), and 600 ng of Cy3-labeled cRNA was fragmented and hybridized to a SurePrint G3 Gene Expression Microarray at 65 °C for 17 hours. Then, the microarray was scanned using an Agilent G2565BA microarray scanner (Agilent Technologies). The obtained signals were processed by Feature Extraction Ver.9.1 (Agilent Technologies), and analyzed by GeneSpring Ver.12.5 (Agilent Technologies). The signal intensity of each probe was normalized so that the 75th percentile of signal intensity of all the probes would be 1.0, and the mean signal intensity of all the probes within a specific gene was used as an expression level of the gene. Gene expression data of mesenchymal stem cells (n = 7, GSE28974) and osteoblasts (n = 3, GSE33382) were obtained from GEO.

### Quantitative methylation-specific PCR (qMSP)

One μg of DNA was treated with sodium bisulfite using an EZ DNA Methylation Kit (Zymo Research, CA). Modified DNA was purified using a Zymospin column I (Zymo Research), and eluted with 40 μl of 1 × TE. One μl of bisulfite-modified DNA was used for quantitative PCR using primers in Supplementary Table 6 and known numbers of molecules of standard DNA. DNA methylation levels were calculated as the percentage of the methylation reference (PMR) [(number of methylated molecules at a target CGI in a sample)/(number of Alu repeat sequences in the sample)]/[(number of methylated molecules at the target CGI in a fully methylated DNA)/(number of Alu repeat sequences in the fully methylated DNA)] × 100^53,54^. Fully methylated DNA was prepared by treating genomic DNA with *SssI* methylase (New England Biolabs, Beverly, MA). Alu repeat sequences were used for normalization of the amount of DNA because their copy number is known to be less affected by cancer-associated aneuploidy and copy number changes, compared with single-copy genes^53^.

### Gene ontology analysis

Enrichment of specific biological processes in gene ontology criteria among genes whose methylation status were changed was analyzed using DAVID bioinformatics resources^55,56^.

### *In vivo* 5-aza-2′-deoxycytidine treatment of xenograft tumors

MNNG/HOS or 143B cells were inoculated into 5-week-old female BALB/c-nu/nu mice (Charles River, Yokohama, Japan). 5-Aza-dC was intraperitoneally administrated at 0, 1 and 2 mg/kg body weight (three consecutive days and 4 days off, 3 and 4 cycles for the MNNG/HOS and 143B xenograft, respectively). The length and width of tumors were measured with standard calipers twice per week and tumor volumes calculated using the formula: tumor volume = (length × width^2^) × 0.5. At sacrifice, the xenograft tissues were measured and histologically analyzed.

### Statistical analysis

Statistical analyses were performed using the PASW Statistics 18 package (SPSS, Chicago, IL, USA). Significance in the difference between two groups was evaluated with the Student t-test and chi-square test. Disease-free survival curves were plotted according to the Kaplan-Meier method, and the log-rank test was applied for comparison. All differences at the level of *P* < 0.05 by a two-sided test were considered statistically significant.

## Supplementary information

Supplementary Information.

## Data Availability

The data of genome-wide analysis of DNA methylation, and gene expression were submitted to the Gene Expression Omnibus (GEO) database under accession no. GSE125645. The other original data are available upon request.
