# Supplementary material for: Epigenetic reprogramming underlies efficacy of DNA demethylation therapy in osteosarcomas
Source: Sci Rep. 2019 Dec 30;9:20360. doi: 10.1038/s41598-019-56883-0 (PMC6937291; doi:10.1038/s41598-019-56883-0)
Supplement: Supplementary file 1 — Supplementary Information. [file 41598_2019_56883_MOESM1_ESM.docx]

**Supplementary Information**

# Epigenetic reprogramming underlies efficacy of DNA demethylation therapy in osteosarcomas

Naofumi Asano^1,2^, Hideyuki Takeshima^3^, Satoshi Yamashita^3^, Hironori Takamatsu^2, 3^, Naoko Hattori^3^, Takashi Kubo^4^, Akihiko Yoshida^5^, Eisuke Kobayashi^6^, Robert Nakayama^2^, Morio Matsumoto^2^, Masaya Nakamura^2^, Hitoshi Ichikawa^4^, Akira Kawai^6^, Tadashi Kondo^1^, and Toshikazu Ushijima^3^

^1^Division of Rare Cancer Research, National Cancer Center Research Institute, 5-1-1 Tsukiji, Chuo-ku, Tokyo 104-0045, Japan; ^2^Department of Orthopaedic Surgery, Keio University School of Medicine, 35 Shinanomachi, Shinjuku-ku, Tokyo 160-8582, Japan; ^3^Division of Epigenomics, National Cancer Center Research Institute, 5-1-1 Tsukiji, Chuo-ku, Tokyo 104-0045, Japan; ^4^Department of Clinical Genomics, National Cancer Center Research Institute, 5-1-1 Tsukiji, Chuo-ku, Tokyo 104-0045, Japan; ^5^Department of Pathology and Clinical Laboratory, National Cancer Center Hospital, 5-1-1 Tsukiji, Chuo-ku, Tokyo 104-0045, Japan; ^6^Department of Musculoskeletal Oncology, National Cancer Center Hospital, 5-1-1 Tsukiji, Chuo-ku, Tokyo 104-0045, Japan

**Supplementary Information**

**Supplementary Figure 1.** The *in vivo* effect of the DNA methylation treatment on 143B cells.

**Supplementary Table 1.** Clinical characteristics of 31 OS and 11 EWS patients

**Supplementary Table 2.** Genes analyzed for mutations (NCC Oncopanel v4)

**Supplementary Table 3.** Univariate analysis of factors influencing progression-free survival (PFS) in the 24 patients with primary localized OS

**Supplementary Table 4.** Re-expression of 31 genes by demethylation of MG63 cell

**Supplementary Table 5.** Re-expression of 13 genes by demethylation of U2OS cell

**Supplementary Table 6.** Primer sets used for quantitative Methylation-Specific PCR (qMSP)


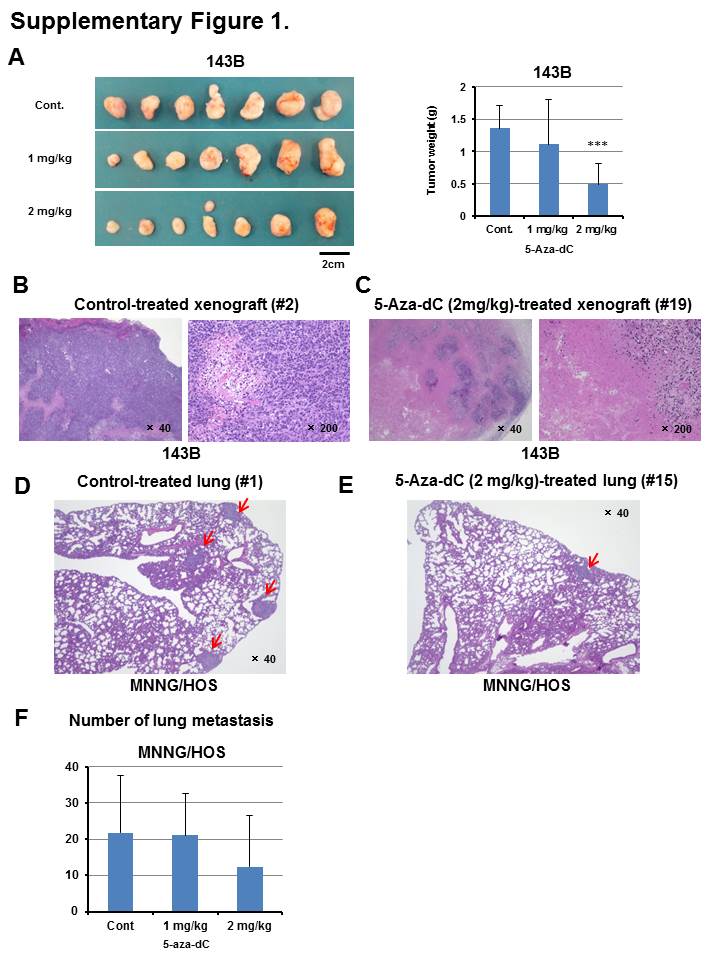


**Supplementary Figure 1.** The *in vivo* effect of the DNA methylation treatment on 143B cells. (**A**) The treatment of 143B cells with 5-aza-dC reduced the tumor volume and weight of 143B xenograft tumors. (**B - F**) Pathological analysis of the hematoxylin-stained specimens. In the 5-aza-dC-treated tumors, marked tumor cell necrosis of 143B tumor xenograft (**C**) and reduction of lung metastasis of MNNG/HOS xenograft (**F**, and **G**) was observed as compared with the non-administration tumor (**B**, and **D**).

**Supplementary Table 1. Clinical characteristics of 31 OS and 11 EWS patient**

|  | **Number of patients** | | |
| --- | --- | --- | --- |
| **Variable** | **Total (N=42)** | **OS (N=31)** | **EWS (N=11)** |
| **Age (years)** |  |  |  |
| **Median (range)** | 15 (7-68) | 13 (7-68) | 19 (9-65) |
| **Sex** |  |  |  |
| **Male** | 24 | 16 | 8 |
| **Female** | 18 | 15 | 3 |
| **Primary tumor site** |  |  |  |
| **Extremity** | 33 | 27 | 5 |
| **Trunk** | 9 | 4 | 6 |
| **Primary tumor origin** |  |  |  |
| **Bone** | 37 | 31 | 6 |
| **Soft tissue** | 5 | 0 | 5 |
| **Primary tumor size (cm)** |  |  |  |
| **Median (range)** | 9.5 (4.0 - 20.5) | 10.0 (4.0 - 20.5) | 9.0 (4.3 - 20.0) |
| **M0/M1** |  |  |  |
| **M0** | 38 | 28 | 10 |
| **M1** | 4 | 3 | 1 |
| **TNM Stage (UICC 7^th^ ed.)** |  |  |  |
| **IA/IB** | 0 | 0 | 0 |
| **IIA/IIB** | 33 | 26 | 7 |
| **III** | 5 | 2 | 3 |
| **IV** | 4 | 3 | 1 |
| **Treatment** |  |  |  |
| **Surgery + chemo** | 33 | 27 | 6 |
| **Surgery + chemo + RT** | 2 | 0 | 2 |
| **RT or CIRT + chemo** | 5 | 3 | 2 |
| **Palliative chemo therapy** | 2 | 1 | 1 |
| **Local recurrence** |  |  |  |
| **no** | 35 | 26 | 9 |
| **yes** | 3 | 2 | 1 |
| **NE** | 4 | 3 | 1 |
| **Distant metastasis** |  |  |  |
| **no** | 22 | 17 | 5 |
| **yes** | 16 | 11 | 5 |
| **NE** | 4 | 3 | 1 |
| **Recurrence** |  |  |  |
| **no** | 20 | 15 | 5 |
| **yes** | 18 | 13 | 5 |
| **NE** | 4 | 3 | 1 |
| **Follow up (months)** |  |  |  |
| **Median (range)** | 63.3 (3.8 – 240.1) | 68.9 (3.8 - 240.1) | 37.6 (17.8 - 145.6) |
| **Oncological outcome** |  |  |  |
| **No evidence of disease** | 22 | 18 | 4 |
| **Alive with disease** | 6 | 4 | 1 |
| **Dead of disease** | 13 | 9 | 5 |
| **Dead of others** | 1 | 0 | 1 |

chemo, chemotherapy; RT, radiation therapy; CIRT, carbon ion radiation therapy; NE, not evaluable

**Supplementary Table 2. Genes analyzed for mutations (NCC Oncopanel v4)**

| **For somatic mutations and copy number alterations** | | | |  |  | **For fusions** |
| --- | --- | --- | --- | --- | --- | --- |
| *ABL1* | *CRKL* | *IDH2* | *NF1* | *RAC2* |  | *ALK* |
| *ACTN4* | *CREBBP* | *IGF1R* | *NFE2L2* | *RAD51C* |  | *AKT2* |
| *AKT1* | *CTNNB1* | *IGF2* | *NOTCH1* | *RAF1* |  | *BRAF* |
| *AKT2* | *CUL3* | *IL7R* | *NOTCH2* | *RB1* |  | *ERBB4* |
| *AKT3* | *DDR2* | *JAK1* | *NOTCH3* | *RET* |  | *FGFR2* |
| *ALK* | *EGFR* | *JAK2* | *NRAS* | *RHOA* |  | *FGFR3* |
| *APC* | *ENO1* | *JAK3* | *NRG1* | *ROS1* |  | *NRG1* |
| *ARAF* | *EP300* | *KDM6A* | *NTRK1* | *SETBP1* |  | *NTRK1* |
| *ARID1A* | *ERBB2* | *KEAP1* | *NTRK2* | *SETD2* |  | *NTRK2* |
| *ARID2* | *ERBB3* | *KIT* | *NTRK3* | *SMAD4* |  | *PDGFRA* |
| *ATM* | *ERBB4* | *KRAS* | *NT5C2* | *SMARCA4* |  | *RET* |
| *AXIN1* | *ESR1* | *MAP2K1* | *PALB2* | *SMARCB1* |  | *ROS1* |
| *AXL* | *EZH2* | *MAP2K2* | *PBRM1* | *SMO* |  |  |
| *BAP1* | *FBXW7* | *MAP2K4* | *PDGFRA* | *STAT3* |  |  |
| *BARD1* | *FGFR1* | *MAP3K1* | *PDGFRB* | *STK11* |  |  |
| *BCL2L11* | *FGFR2* | *MAP3K4* | *PIK3CA* | *TP53* |  |  |
| *BRAF* | *FGFR3* | *MDM2* | *PIK3R1* | *TSC1* |  |  |
| *BRCA1* | *FGFR4* | *MDM4* | *PIK3R2* | *VHL* |  |  |
| *BRCA2* | *FLT3* | *MET* | *POLD1* |  |  |  |
| *CCND1* | *GNA11* | *MLH1* | *POLE* |  |  |  |
| *CD274* | *GNAQ* | *MTOR* | *PRKCI* |  |  |  |
| *CDK4* | *GNAS* | *MSH2* | *PTCH1* |  |  |  |
| *CDKN2A* | *HRAS* | *MYC* | *PTEN* |  |  |  |
| *CHEK2* | *IDH1* | *MYCN* | *RAC1* |  |  |  |

| **Variable** | **Number of cases** | **Univariate** | |
| --- | --- | --- | --- |
|  |  | **5y-PFS rate (%)** | ***P* value** |
| **Methylation cluster** |  |  |  |
| **cluster III-a** | 14 | 75.0 | 0.361 |
| **cluster IV-a** | 10 | 50.0 |  |
| ***TP53* gene alteration** |  |  |  |
| **Yes** | 9 | 88.9 | 0.064 |
| **No** | 15 | 53.3 |  |
| **Age** |  |  |  |
| **< 18 years** | 19 | 78.9 | 0.008 |
| ≥ **18 years** | 5 | 20.0 |  |
| **Sex** |  |  |  |
| **Male** | 13 | 61.5 | 0.437 |
| **Female** | 11 | 72.7 |  |
| **Tumor site** |  |  |  |
| **Extremity** | 21 | 71.4 | 0.007 |
| **Trunk** | 3 | 33.3 |  |
| **Tumor size** |  |  |  |
| **≤ 8cm** | 3 | 66.7 | 0.707 |
| **> 8cm** | 21 | 66.7 |  |
| **Histological response to chemotherapy** |  |  |  |
| **Good (< 10% viable cells)** | 11 | 90.9 | 0.045 |
| **Poor (**≥ **10% viable cells)** | 10 | 50.0 |  |

**Supplementary Table 3. Univariate analysis of factors influencing progression-free survival (PFS) in the 24 patients with primary localized OS***

*We excluded from survival analysis one patient whose follow up term was only 3.8 months. OS, osteosarcoma; HR, hazard ratios; 95% CI, 95% confidence interval; NS, not significant

**Supplementary Table 4. Re-expression of 31 genes by demethylation of MG63 cells**

| **Gene symbol** | **Gene Name** | **Gene function** |
| --- | --- | --- |
| ***HSPA2*** | heat shock 70kDa protein | expression in cancer cells |
| ***NETO2*** | neuropilin (NRP) and tolloid (TLL)-like 2 | endocytosis |
| ***GREM1^a, b^*** | gremlin 1, DNA family BMP antagonist | methylation in cancers (tumor suppressor), bone formation |
| ***QPCT^a^*** | glutaminyl-peptide cyclotransferase | methylation in melanoma (tumor suppressor) |
| ***IGFBP2^b^*** | insulin-like growth factor | cancer cell proliferation, bone formation, osteoblast function |
| ***PRSS23*** | protease, serine, 23 | NA |
| ***LOC93622*** | Morf4 family associated protein 1-like 1 | NA |
| ***IRS2*** | insulin receptor substrate 2 | tumorigenesis, metastasis |
| ***FNDC1*** | fibronectin type III domain containing 1 | tumor growth, metastasis |
| ***ULBP1^a^*** | UL16 binding protein 1 | activate human natural killer (NK) cells |
| ***PPL*** | periplakin | tumor progression |
| ***TES^a^*** | testis derived transcript (3 LIM domains) | methylation in ovarian carcinoma (tumor suppressor) |
| ***PTGER4^b^*** | prostaglandin E receptor 4 (subtype EP4) | bone formation |
| ***FOXQ1^b^*** | forkhead box Q1 | overexpression in colorectal and lung adenocarcinoma, embryogenesis |
| ***DSP*** | desmoplakin | cancer cell proliferation, cell adhesion |
| ***TLE4*** | transducin-like enhancer of split 4 | B cell differentiation, |
| ***SH2D4A*** | SH2 domain containing 4A | NA |
| ***GCHFR*** | GTP cyclohydrolase I feedback regulator | expression in prostate cancer, oxidant-induced cell death |
| ***EFNB2*** | ephrin-B2 | expression in cancers |
| ***DLX5^b^*** | distal-less homeobox 5 | bone morphogenesis, chondrogenesis, embryonic development, tooth morphogenesis |
| ***STC2^b^*** | stanniocalcin 2 | bone development |
| ***PLTP*** | phospholipid transfer protein | DM, HL |
| ***FBXO6*** | F-box protein 6 | differentiation hepatocytes |
| ***MBP*** | myelin basic protein | demyelination, T cell-mediated autoimmunity, regulation of CXCL12 expression in astrocytes by soluble MBP |
| ***KLF4^a^*** | Kruppel-like factor 4 (gut) | suppressing the tumorigenicity of colon cancer cells, p53-dependent cell cycle arrest |
| ***CLDN11*** | claudin 11 | oligodendrocyte morphogenesis, cell differentiation |
| ***FMN2*** | formin 2 | expression in cancers |
| ***CYBA*** | cytochrome b-245, alpha polypeptide | chronic granulomatous disease, NADPH oxidase |
| ***EPSTI1*** | epithelial stromal interaction 1 | epithelial-stromal interaction in breast cancer |
| ***CXCL12^a^*** | chemokine (C-X-C motif) ligand 12 | hematopoiesis, T cell activation |
| ***GMPR*** | guanosine monophosphate | neoplastic or normal cell proliferation |

a, osteo/chondrogenesis related genes; b, tumor-suppressor genes

**Supplementary Table 5. Re-expression of 13 genes by demethylation of U2OS cells**

| **Gene symbol** | **Gene Name** | **Gene function** |
| --- | --- | --- |
| ***QPCT^a^*** | glutaminyl-peptide cyclotransferase | methylation in melanoma (tumor suppressor) |
| ***TNFRSF10D^a^*** | tumor necrosis factor receptor superfamily 10D | OS cells Apo2L/TRAIL-induced apoptosis, promoter hypermethylation in neuroblastoma |
| ***FNDC1*** | fibronectin type III domain containing 1 | tumor growth, metastasis |
| ***SPINT2^a^*** | serine peptidase inhibitor, Kunitz type 2 | tumor suppressor gene, promoter region methylation |
| ***PPL*** | periplakin | tumor progression |
| ***CST6^a^*** | cystatin E/M | tumor suppressor gene, epigenetic inactivation |
| ***CTHRC1*** | collagen triple helix repeat containing 1 | cancer cell migration |
| ***MBP*** | myelin basic protein | demyelination, T cell-mediated autoimmunity, regulation of CXCL12 expression in astrocytes by soluble MBP |
| ***CPT1C*** | carnitine palmitoyltransferase 1C | NA |
| ***GPR68^a, b^*** | G protein-coupled receptor 68 | termed ovarian cancer G protein-coupled receptor 1 (OGR1), RANKL-induced osteoclast differentiation, IFN-alpha-activated NK cells |
| ***ALPL^a, b^*** | alkaline phosphatase, liver/bone/kidney | tumor suppressor gene, bone mineralization |
| ***COMP^b^*** | cartilage oligomeric matrix protein | differentiated chondrocyte |
| ***COL15A1*** | collagen, type XV, alpha 1 | dermal fibroblast |

a, osteo/chondrogenesis related genes; b, tumor-suppressor genes

**Supplementary Table 6. Primer sets used for qMSP**

| ***TSPYL5* forward** |
| --- |
| GATTTTGAGAGTACGATGAGTTC |
| ***TSPYL5* reverse** |
| CGAACTCGCTTTAAATCTCGACG |
|  |
| ***TNFRSF10D* forward** |
| GTCGGAAAGAGTTAGTTTTTGTTC |
| ***TNFRSF10D* reverse** |
| AATAAAACGTACTCCTCCG |
|  |
| **Alu N forward** |
| GGTTAGGTATAGTGGTTTATATTTGTAATTTTAGTA |
| **Alu N reverse** |
| ATTAACTAAACTAATCTTAAACTCCTAACCTCA |
